# Species Delimitation of the *Cycas segmentifida* Complex (Cycadaceae) Resolved by Phylogenetic and Distance Analyses of Molecular Data

**DOI:** 10.3389/fpls.2016.00134

**Published:** 2016-02-15

**Authors:** Xiuyan Feng, Jian Liu, Xun Gong

**Affiliations:** ^1^Key Laboratory for Plant Diversity and Biogeography of East Asia, Kunming Institute of Botany, Chinese Academy of SciencesKunming, China; ^2^University of Chinese Academy of SciencesBeijing, China

**Keywords:** *Cycas segmentifida* complex, delimitation, phylogeny, species, synonym

## Abstract

The *Cycas segmentifida* complex consists of eight species whose distributions overlap in a narrow region in Southwest China. These eight taxa are also morphologically similar and are difficult to be distinguished. Consequently, their taxonomic status has been a matter of discussion in recent years. To study this species complex, we sequenced four plastid intergenic spacers (cpDNA), three nuclear genes and genotyped 12 microsatellites for the eight taxa from 19 different localities. DNA sequences were analyzed using Maximum Likelihood (ML) method and Bayesian Inference (BI), and microsatellites were analyzed using the Neighbor-joining (NJ) and structure inference methods. Results of cpDNA, nuclear gene *GTP* and microsatellites all rejected the hypotheses that this complex consisted of eight taxa or one distinct lineage (species) but two previously described species were adopted: *Cycas guizhouensis* K. M. Lan et R. F. Zou and *Cycas segmentifida* D. Y. Wang et C. Y. Deng. *Cycas longlinensis* H. T. Chang et Y. C. Zhong was included in *C. guizhouensis* and the other five taxa were included in *C. segmentifida*. Our species delimitation inferred from molecular data largely corresponds to morphological differentiation. However, the other two nuclear genes were unable to resolve species boundaries for this complex independently. This study offered evidences from different genomes for dealing with the species boundaries and taxonomical treatment of the *C. segmentifida* complex in an integrated perspective.

## Introduction

Species delimitation is essential since species is regarded as the basic unit of analysis in nearly all biological disciplines, such as ecology, biogeography, conservation biology, and macroevolution (Mayr, 1982). Any incorrect species delimitation may result in more serious errors in succeeding relevant studies, which will increase the costs of species conservation or lead to an unpredictable waste of effort (Wiens, 2007). From a common accepted view, a species is considered as an evolutionarily distinct lineage which can be distinguished from other lineages due to reproductive isolation and termination of gene flow, niche differentiation and other lines of evidence (De Queiroz, 1998, 2007; Stockman and Bond, 2007; Bond and Stockman, 2008; Fujita et al., 2012; Hendrixson et al., 2013; Mckay et al., 2013). The methods of species delimitation mainly have two general categories: tree-based and non-tree-based approaches (Sites and Marshall, 2003, 2004). Delimiting species by tree-based methods are carried out by recognizing species as distinguishing clades (phylogenetic species concept), whereas delimitation species by non-tree-based methods are implemented on the basis of gene flow assessments (biological species concept; Pérez-Losada et al., 2005). However, few specific species delimitation criteria have been proposed, within which Wiens and Penkrot presented and compared three specific criteria that were based on DNA data, morphological data and character data respectively to achieve species delimitation validly, which provided a key theoretical frame for empirical studies (Wiens and Penkrot, 2002).

*Cycas guizhouensis* K. M. Lan et R. F. Zou (Lan and Zou, 1983) and *Cycas segmentifida* D. Y. Wang et C. Y. Deng (Wang and Deng, 1995) were two primarily published *Cycas* species from Guizhou province of China, whereas six similar taxa, *Cycas longlinensis* H. T. Chang et Y. C. Zhong (Zhang and Zhong, 1997), *Cycas multifida* H. T. Chang et Y. C. Zhong (Zhang et al., 1997), *Cycas crassipes* H. T. Chang, Y. Z. Zhong et Z. F. Lu (Zhang et al., 1999), *Cycas xilingensis* H. T. Chang et Y. C. Zhong (Zhang and Zhong, 1997), *Cycas longiconifera* H. T. Chang, Y. C. Zhong et Y. Y. Huang (Zhang et al., 1997), and *Cycas acuminatissima* H. T. Chang, Y. C. Zhong et Z. F. Lu (Zhang et al., 1997) were subsequently described, constituting the *C. segmentifida* complex which are all endemic to Southwest China, mainly in southwestern Guizhou, northwestern Guangxi and eastern Yunnan province. It is the complicated terrain, diversified climates and habitats that result in a high degree of diversification in the genus *Cycas* in this area, which even makes one continuously distributed species in different regions exhibiting different morphologies. *Cycas guizhouensis* is widely distributed in the valleys of the Nanpan River characterized by fusiform male cone, loose and open female cone, densely hariy sporophylls, nearly round apical lobe (the margins with numerous tapered lobes) and yellow with reddish brown mucro seeds (nearly globose; Jones, 1998). *Cycas longlinensis* only exists in Jinzhongshan, Longlin, Guangxi province and is characterized by narrower and longer pinnae, and fewer and broader segments in microsporophyll (Zhang and Zhong, 1997). It resembles *C. guizhouensis* in morphology, and the above two species have an overlapped distribution along the Nanpan River. *Cycas multifida* is distinguished by its numerous and glabrous lateral segments in macrosporophyll and distributed only in Bada, Xilin, Guangxi province (Zhang and Zhong, 1997). *Cycas xilingensis* is distinct from other taxa by its thinner and longer carpophyll and its much longer and wider terminal segment (Zhang and Zhong, 1997). *Cycas crassipes* is distinguished by its robust pedicels (Zhang et al., 1999) with only one population in Bianya, Longlin, Guangxi province. *Cycas segmentifida* is widespread, mainly distributed in southwestern Guizhou, northwestern Guangxi and eastern Yunnan province along the You River, and can be characterized by its usually pruinose annal petiole, dichotomus or sometimes forked, acuminate, aristate apically of lateral segments, and ovate-retunded terminal sterile lamina which is covered with caducous brown-tomentose (Wang and Deng, 1995). *Cycas longiconifera* is mainly distributed in the Baise, Guangxi province and can be distinguished by its slenderly cylindrical male cone (Zhang et al., 1997). *Cycas acuminatissima* is characterized by its pinnae with acuminate apex and shorter carpophylls (Zhang et al., 1997) and is distributed in northwestern Guangxi province.

The taxonomic status of these similar taxa within the *C. segmentifida* complex has been a matter of debate in recent years. The *Flora of China* (Chen and Stevenson, 1999) treated *Cycas acuminatissima, C. longlinensis, C. multifida*, and *C. xilingensis* in the synonymy of *Cycas segmentifida*, while treated *Cycas guizhouensis* in the synonymy of *Cycas szechuanensis*, and subsequently Wang (2000) proposed to classify *C. longlinensis* into *C. guizhouensis*, the remaining five taxa into *C. segmentifida* and considered the complex containing only two valid species (i.e., *C. guizhouensis* and *C. segmentifida*). Huang (2001) accepted these eight taxa based on their morphological character comparisons, while Whiteloek (2002) placed *C. longiconifera, C. multifida*, and *C. xilingensis* into *C. segmentifida*, put *C. acuminatissima* into *C. sexseminifera* and *C. longlinensis* into *C. guizhouensis*. The World List of Cycads Group placed *Cycas acuminatissima, C. longlinensis, C. multifida*, and *C. xilingensis* in the synonymy of *Cycas segmentifida* and accepted *C. guizhouensis* as a distinct taxon (Calonje et al., 2015; http://cycadlist.org/index.php). A recent study which combined morphology and ISSR methods suggested that the *C. segmentifida* complex only contained two species with *C. longlinensis* incorporated into *C. guizhouensis* while the remaining five species into *C. segmentifida* (Ma, 2005). At present, the classification of these taxa is not fully settled. According to field investigations, we found that *C. longlinensis* had no substantial difference with *C. guizhouensis* in morphology. The remaining five taxa were sympatric with *C. segmentifida*. *Cycas guizhouensis* and *C. segmentifida* belonged to two different basins (Nanpan vs. You River) and could be easily distinguished by their leaves and megasporophylls. In this study, species delimitation was carried out on all the above eight taxa by using DNA sequences and microsatellite markers with a tree-based haplotype aggregation methods and genetic structure inference, aiming to deal with their boundaries from a genetic and phylogenetic perspective. With the above methods, we propose the following possible hypotheses for the present taxa:

1. Eight independent clades would be identified according to the evidence from either DNA haplotypes or genetic structure inferred by SSR data.
2. None of the eight taxa could be distinguishable while only one single lineage was formed because of complete lineage sorting or strong gene flow and natural hybridization in this area.
3. At least two distinct groups which corresponded to morphology and geography can be identified.

## Materials and methods

### Cycas *segmentifida* complex sampling

**Figure 1:**
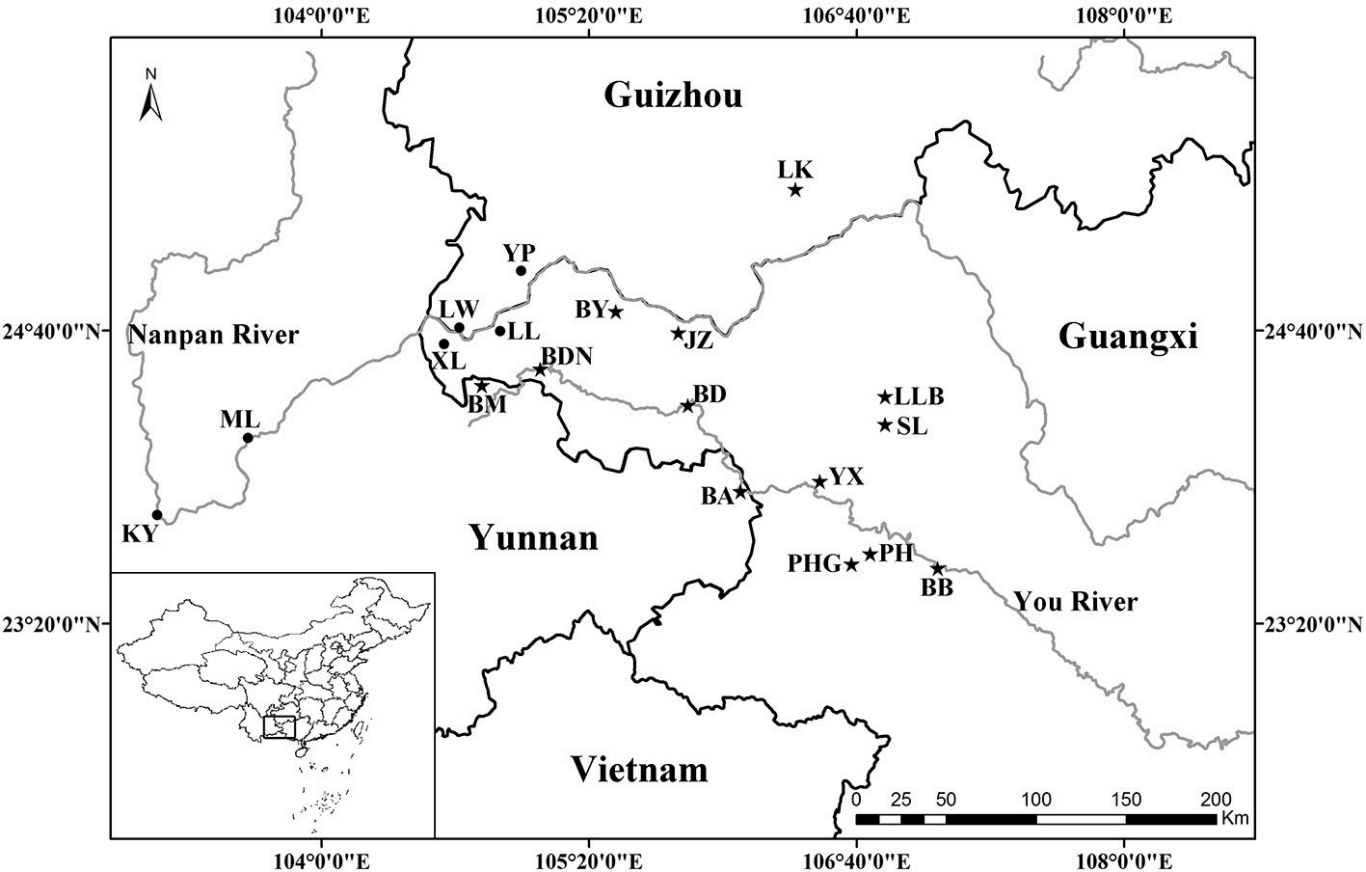
**Geographical distribution of 19 populations of the *C. segmentifida* complex**. Maps were drawn using the software ArcGIS version 10.2 (http://desktop.arcgis.com).

All known species in the *C. segmentifida* complex were investigated and sampled in this study. A total of 311 individuals from eight taxa of this complex were collected from 19 different populations in southwestern Guizhou, northwestern Guangxi and eastern Yunnan province of Southwest China. Young and healthy leaves were dried in silica gel immediately after collection. Within the 311 samples, 5 individuals from each location, except for population BDN where only two individuals were found, were randomly selected for plastid and nuclear DNA sequencing while all the 311 individuals were used for the microsatellite study. Sampling information on each *C. segmentifida* complex species is summarized in Table 1 and Figure 1.

**Table 1:** **Details of sample locations, sample sizes (n) surveyed for DNA sequences and microsatellites of eight taxa of the *C. segmentifida* complex**.

**Species**	**Sample sites; Code; Individuals for DNA sequences and microsatellites**	**Latitude N°**	**Longitude E°**	**Altitude m**
*C. longlinensis*	Longlin, Guangxi; LL; 5/20	24.665	104.889	970
*C. guizhouensis*	Yangping, Xingyi, Guizhou; YP; 5/20	24.938	104.994	930
	Luowan, Xingyi, Guizhou; LW; 5/20	24.681	104.686	880
	Xilin, Guangxi; XL; 5/20	24.606	104.611	860
	Mile, Yunnan; ML; 5/20	24.181	103.632	1430
	Kaiyuan, Yunnan; KY; 5/20	23.829	103.180	1400
*C. multifida*	Bada, Xilin, Guangxi; BDN; 2/2	24.492	105.091	750
*C. crassipes*	Bianya, Longlin, Guangxi; BY; 5/20	24.754	105.468	560
*C. xilingensis*	Jiuzhou, Tianlin; Guangxi; JZ; 5/10	24.657	105.780	490
*C. segmentifida*	Lekuan, Wangmo, Guizhou; LK; 5/20	25.304	106.363	650
	Boai, Funing, Yunnan; BA; 5/20	23.936	106.090	300
	Bamei, Guangnan, Yunnan; BM; 5/20	24.418	104.897	960
	Badu, Tianlin, Guangxi; BD; 5/15	24.327	105.827	300
*C. longiconifera*	Yangxu, Baise, Guangxi; YX; 5/7	23.982	106.485	350
*C. acuminatissima*	Luolou, Lingyun, Guangxi; LLB; 5/12	24.367	106.810	760
	Shali, Lingyun, Guangxi; SL; 5/14	24.241	106.811	490
	Pohong, Tianyang, Guangxi; PH; 5/20	23.652	106.736	570
	Gumei, Pohong, Tianyang, Guangxi; PHG; 5/20	23.605	106.643	660
	Bubing, Tiandong, Guangxi; BB; 5/20	23.586	107.072	150

### DNA extraction, PCR amplification, and genotyping

We extracted genomic DNA from dried leaves using the modified CTAB method (Doyle, 1991). DNA was dissolved in TE buffer and stored at –20°C. After preliminary screening from universal plastid and nuclear primers, four cpDNA intergenic spacers, *psb*A-*trn*H (Chiang and Peng, 1998), *psb*M-*trn*D (Shaw et al., 2005), *trn*S-*trn*G (Shaw et al., 2005), and *trn*L-*trn*T (Taberlet et al., 1991) and three nuclear genes, *GTP*, guanosine triphosphate (GTP) gene (Salas-Leiva et al., 2014); *PHYP*, phytochrome P gene and *PPRC*, hypothetical protein gene (unpublished) were chose for complete analysis (Table 2).

**Table 2:** **Sequences of plastid and nuclear gene primers used in this study**.

**Region**	**Primer sequences (5′—sequence—3′)**	**References**
*psb*A-*trn*H	*psb*A: GTTATGCATGAACGTAATGCTC*trn*H: CGCGCATGGTGGATTCACAAATC	Chiang and Peng, 1998
*psb*M-*trn*D	*psb*M:AGCAATAAATGCRAGAATATTTACTTCCAT*trn*D: GGGATTGTAGYTCAATTGGT	Shaw et al., 2005
*trn*S-*trn*G	*trn*S: GCCGCTTTAGTCCACTCAGC*trn*G: GAACGAATCACACTTTTACCAC	Shaw et al., 2005
*trn*L-*trn*T	*trn*L: TCTACCGATTTCGCCATATC*trn*T: CATTACAAATGCGATGCTCT	Taberlet et al., 1991
*GTP*	*GTP*F: TGATACWCCTGGTGTGAT*GTP*R: CTCCATSTCCATATTTGGC	Salas-Leiva et al., 2014
*PHYP*	*PHYP*F: CCAGTCTCCCAGTATCATGG*PHYP*R: GCTGCATGATATTTCCAACC	This study
*PPRC*	*PPRC*F: CAAAACTATGCTGTCAATCC*PPRC*R: TTAGCATCACCAGTAATCCC	This study

Amplification protocols were as follows: for cpDNA, each 30μL reaction contained 15 ng DNA, 3.0μL 10 × PCR buffer, 1.5μL MgCl_2_ (25mM), 1.5μL dNTPs (10mM), 1.5μL DMSO, 0.45μL of each primer, 0.45μL Taq DNA polymerase (5 U/μL) (Takara, Shiga, Japan) and 19.5μL double-distilled water; for nuclear genes, the PCR reactions contained 30 ng DNA, 3.0μL 10 × PCR buffer, 2.25μL MgCl_2_ (25mM), 2.25μL dNTPs (10mM), 2.25 DMSO, 0.6μL of each primer, 0.5μL Taq DNA polymerase (5 U/μL) (Takara, Shiga, Japan) and 15.53μL double-distilled water. PCR amplifications were performed in a thermocycler under the following conditions: an initial 5 min denaturation at 80°C, followed by 29 cycles of 1 min at 95°C, 1 min annealing at 50°C, and a 1.5 min extension at 65°C, and a final extension for 5 min at 65°C for cpDNA intergenic spacers. For nuclear genes, we used an initial 4 min denaturation at 94°C, which was followed by 34 cycles of 45 s at 94°C, 1 min annealing at 54, 55 and 50°C for *GTP, PHYP* and *PPRC*, and a 1.5 min extension at 72°C, and a final extension for 9 min at 72°C. All PCR products of different DNA fragments were sequenced directly in both directions by the dideoxy chain-termination method, using an ABI 3730XL automated sequencer (made in Applied Biosystems) at Shanghai Meiji Biological Medicine and Technology Co Ltd. The sequencing primers were the same as those used in the amplification reactions. All the sequences were deposited in GenBank with the accession numbers KU240442-KU240491, KT824924-KT824925, KT824931-KT824932, KT824936, KT824941, KT824946-KT824948, KT824951, KT824954-KT824955, KT824960-KT824962.

Microsatellite markers were selected from recently developed nuclear microsatellites in *Cycas* (Cibrián-Jaramillo et al., 2008; Wang et al., 2008; Yang et al., 2008; Li et al., 2009; Zhang et al., 2009, 2010; Ju et al., 2011). PCR amplification was carried out as in the same way as in *C. simplicipinna* (Feng et al., 2014). After preliminary screening microsatellite loci for the *C. segmentifida* complex, the selected microsatellite loci were stained with fluorescent dye at the 5′ end, their PCR products were separated and visualized using an ABI 3730XL automated sequencer at Shanghai Meiji Biological Medicine and Technology Co Ltd, and their profiles were read with the GeneMapper version 4.0, software (Applied Biosystems, Foster City, CA). An individual was declared null (nonamplifying) at a locus and was treated as missing data after two or more amplification failures. Finally, we chose polymorphic microsatellite loci for the *C. segmentifida* complex, if the microsatellite locus had two or more alleles (Table 3).

**Table 3:** **Information of 12 microsatellite primers for species delimitation of the *C. segmentifida* complex**.

**Lucus**	**Primer sequence**	**Repeat motif**	***T*a (°C)**	**GenBank Accession No**.	**References**	***N*_*A*_**	***H*_*E*_**	***H*_*O*_**
Cha02	F:CGAGGAACATCAAGGCTATG R:CCTAGCTTTTGGGAATTAGAC	(CT)_21_	58	EU795697	Zhang et al., 2009	26	0.891	0.627
Cha08	F:CAGGGACCATTGTTTCTAAGG R:ACTTATACATAGGGCTCTAAT	(AG)_10_	54	EU827617	Zhang et al., 2009	23	0.890	0.411
Cy-Tai EST-SSR11	F:GATATTAAAGGCACGGGAG R:TGAAGCTGCTGCATTTGCAT	(CAG)_34_	56	DR062467	Ju et al., 2011	4	0.120	0.106
E001	F:TGGGATTAATATTCCAGAAA R:CGACGAGTCTGATGTAGGTAT	(CA)_10_	52	DR063256	Yang et al., 2008	10	0.332	0.219
E004	F:CTATCATCAGAGCCTCATTC R:AAGTCATACATGGACAGCAA	(AT)_11_	54	DR063135	Yang et al., 2008	10	0.835	0.585
Cpz26	F:GTCCATAATACATATCCACGAA R:GATGATGGCAAACAGAAGC	(AT)_16_	55	EX928897.1	Zhang et al., 2010	17	0.773	0.479
HL08	F:AAAACATTCCTTGCCCTGT R:GGAGCCTGTTGAAGAGCTA	(TTC)_12_	56	EU791556	Li et al., 2009	10	0.402	0.277
CY232	F:TCTTGCTTACCCGTTTGCTT R:CTCCTCGACGTTCAATCACA	(GT)_9_(GCGT)_3_	55	None	Cibrián-Jaramillo et al., 2008	5	0.424	0.309
Cha-estssr01	F:GATTCTTGCTCTGTTCGCTCAT R:CAGAACCCCTGAACTGTCAAAC	(AT)_26_	60	CB091079	Wang et al., 2008	49	0.953	0.342
Cha-estssr02	F:ATAGGCTTCCTTTAGTGATGTC R:GCCTTTAGTAGTATCGGATTA	(CT)_5_(AG)_4_G(GA)_5_	50	DR062468	Wang et al., 2008	7	0.657	0.344
Cha-estssr04	F:GATGTTCCCAAATAATGTTACA R:CAAGCTGCACATGCAATGA	(AT)_3_GT(AT)_9_AG(AC)_4_	54	DR063107	Wang et al., 2008	15	0.872	0.662
Cha05	F:GTCTGCTAACATCTATAAA R:GATGAGCTAAGAGTCATAGTA	(CT)_19_	52	EU795701	Zhang et al., 2009	3	0.056	0.051
Total						179		
Mean						14.917	0.600	0.368

### Sequence processing and phylogenetic reconstruction

Sequences were edited and assembled using SeqMan II (Swindell and Plasterer, 1997). Multiple alignments of DNA sequences were performed manually and subsequently adjusted in Bioedit, version 7.0.4.1 (Hall, 1999). We combined the four cpDNA regions and performed a congruence test in PAUP^*^ 4.0b10 (Swofford, 2003). The testing result showed a significant rate of homogeneity (*P* = 1, >0.5), suggesting a high degree of homogeneity between the four cpDNA regions. The combined cpDNA sequences were therefore used in the next analyses. Three nuclear genes were analyzed separately. Nuclear genes often had heterozygous sites in some individuals, which were identified by overlapping peaks in chromatograms. We resolved the nuclear sequences by applying the algorithms of PHASE (Stephens et al., 2001; Stephens and Donnelly, 2003) in the software package DnaSP, version 5.0 (Librado and Rozas, 2009). The phased nuclear sequences were used in the following analyses. Maps were drawn using the software ArcGIS version 10.2 (http://desktop.arcgis.com).

Haplotypes were inferred from aligned DNA sequences by DnaSP, version 5.0. Phylogenetic relationships among haplotypes were inferred using Maximum Likelihood (ML) and Bayesian Inference (BI) methods with *C. dolichophylla* as the outgroup. The maximum likelihood analysis using Tamura-Nei model and Nearest-Neighbor-Interchange (NNI) ML heuristic method with 1000 bootstrap replications was performed on MEGA, version 5 (Tamura et al., 2011). Bayesian Inference was performed in MrBayes 3.1.2 (Ronquist and Huelsenbeck, 2003). Each Markov chain was started from a random tree and run for 10^6^ cycles with every 100th cycle sampled from the chain. Each analysis was repeated three times for the checking stationarity.

The Wiens and Penkrot's protocol (Wiens and Penkrot, 2002) was used to test species boundaries between haplotypes of the *C. segmentifida* complex. Their approach named a sampling as focal species (the species of interest in the study; here the *C. segmentifida* complex) and nonfocal species (one or more closely related species; *C. dolichophylla*). A haplotype phylogeny tree may show the focal species to be either exclusive or not. The method needs at least two individuals per locality to make the between-population gene flow inferences. It requires a phylogeny of haplotypes (or individuals) of known locality and taxonomic designation. Specific explanations for the method refer to Wiens and Penkrot's article.

### Neighbor-joining analysis and structure inference

Microsatellite data editing and formatting were performed in GenAlEx, version 6.3 (Peakall and Smouse, 2006). Genetic diversity test for microsatellite loci was performed in GenAlEx, version 6.3 by calculating common genetic diversity indices. Input files for other software analysis were exported by GenAlEx, version 6.3. The phylogenetic relationships of sampled species' populations were estimated using Nei's 1983 genetic distance with Neighbor-joining method (NJ) performed in Powermarker, version 3.25 (Lui and Muse, 2005). Confidence in the resulting relationships was assessed using the bootstrap procedure with 1000 bootstrap replicates. The consensus tree was obtained by the procedure consense in the software package Phylip, version 3.68 (Felsenstein, 2002). Genetic structure inference on the microsatellite data was conducted by STRUCTURE, version 2.2 (Pritchard et al., 2000). The combination of an admixture and a correlated-allele frequencies model was used for the analysis. The simulation was run with values of K from 1 to 20 and repeated 20 times for each set. Each run included a burn-in of 1 × 10^5^ iterations and 1 × 10^5^ subsequent MCMC steps. The best-fit number of grouping was evaluated using ΔK and log-likelihood value by STRUCTURE HARVESTER, version 0.6.8 (Earl, 2012).

## Results

### Haplotypes distribution in the *Cycas segmentifida* complex

The four combined plastid DNA regions surveyed across the 92 individuals (19 populations, Table 1) of the *C. segmentifida* complex were aligned, with a total length of 3198 bp. 11 haplotypes (comH1-comH11) were inferred from the cpDNA matrix in total. Of those, plastid haplotype comH1 was shared by *C. longlinensis* and *C. guizhouensis*, comH6 was shared by *C. multifida, C. crassipes, C. xilingensis*, and *C. segmentifida* and the remaining plastid haplotypes were specific to single species with plastid haplotypes comH2-5 specific to *C. guizhouensis*, comH7 unique for *C. longiconifera* and comH8-11 only fixed in *C. acuminatissima*. The aligned nuclear gene *GTP* had a length of 574 bp identifying five haplotypes (comG1-comG5), of which haplotype comG1 was shared by *C. longlinensis* and *C. guizhouensis* while comG3 was shared by the remaining six taxa. 12 haplotypes (comP1-comP12) were derived from the nuclear gene *PHYP*, which shared a consensus length of 930 bp. Of those, the most abundant haplotype comP1 occupied seven taxa except *C. multifida*, while the more frequent haplotype comP2 was predominant in *C. longlinensis* and *C. guizhouensis* and comP5 in the remaining six taxa. The nuclear gene *PPRC* fixed 11 haplotypes (comR1-comR11) with a unified length of 718 bp. Haplotype comR1 and comR3 were the most widely distributed haplotypes, separately occupied *C. longlinensis* and *C. guizhouensis* and the remaining six taxa. The information of plastid haplotypes and nuclear haplotypes and their distribution components in the *C. segmentifida* complex are shown in Table 4.

**Table 4:** **Composition of haplotypes in populations of the *C. segmentifida* complex derived from combined plastid DNA and nuclear genes**.

**Species**	**Pop code**	**Haplotypes**			
		**cpDNA**	***GTP***	***PHYP***	***PPRC***
*C. longlinensis*	LL	comH1	comG1, G2	comP1, P2	comR1
*C. guizhouensis*	YP	comH1	comG1	comP1, P2	comR1, R2
	LW	comH1	comG1	comP1, P2	comR1
	XL	comH1, H2	comG1	comP1, P2	comR1, R2
	ML	comH3	comG1	comP1, P2	comR1, R2
	KY	comH4, H5	comG1	comP1-P4	comR1, R2
*C. multifida*	BDN	comH6	comG3	comP5	comR3
*C. crassipes*	BY	comH6	comG3	comP1, P5	comR3, R4
*C. xilingensis*	JZ	comH6	comG3	comP1, P5	comR3
*C. segmentifida*	LK	comH6	comG3	comP1, P6	comR3
	BA	comH6	comG3	comP1, P7	comR3, R5
	BM	comH6	comG3	comP1, P5	comR3
	BD	comH6	comG3	comP1, P5-P7,	comR3
*C. longiconifera*	YX	comH7	comG3, G4	comP1, P5, P8	comR3, R5-R8
*C. acuminatissima*	LLB	comH8	comG3	comP1, P5-P7	comR5, R7, R8
	SL	comH9	comG3	comP1, P7, P9	comR5-R7
	PH	comH9	comG3, G5	comP1, P6, P10	comR3, R5, R7-R9
	PHG	comH10	comG3, G4	comP1, P5, P7, P11	comR3, R5-R7, R10, R11
	BB	comH9, H11	comG3, G5	comP1, P5, P10, P12	comR3, R5, R6

### Phylogeny of haplotypes

**Figure 2:**
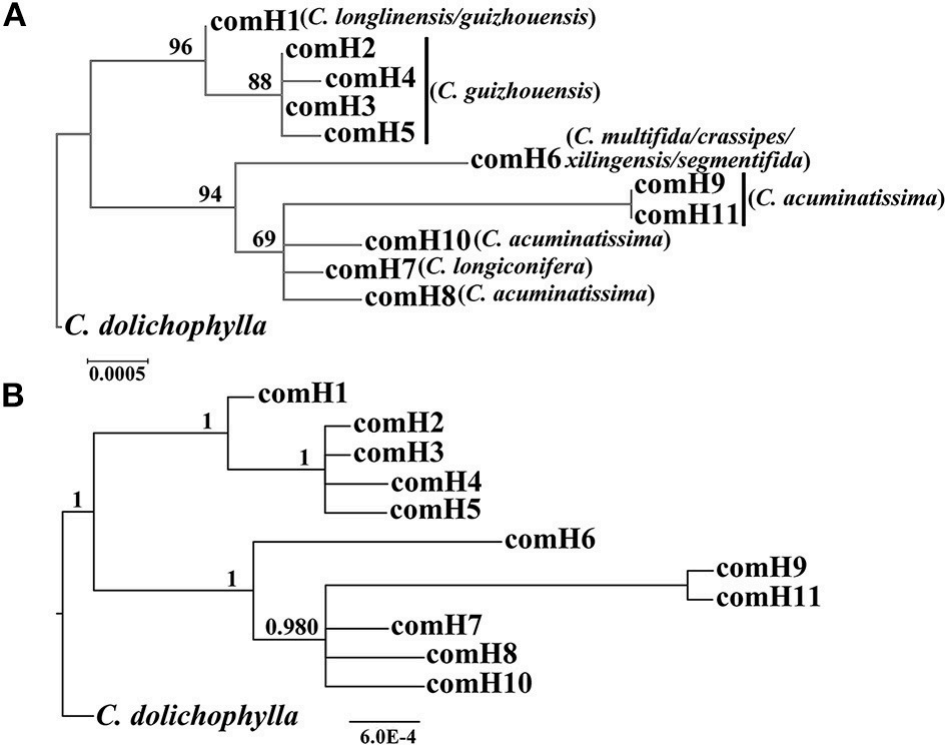
**Phylogenetic trees of plastid haplotypes based on (A) Maximum Likelihood (ML) and (B) Bayesian Inference (BI) methods**. The number (if ≥ 50 or 0.5) on each branch indicates the **(A)** bootstrap value (BS) and **(B)** posterior probability (PP).

**Figure 3:**
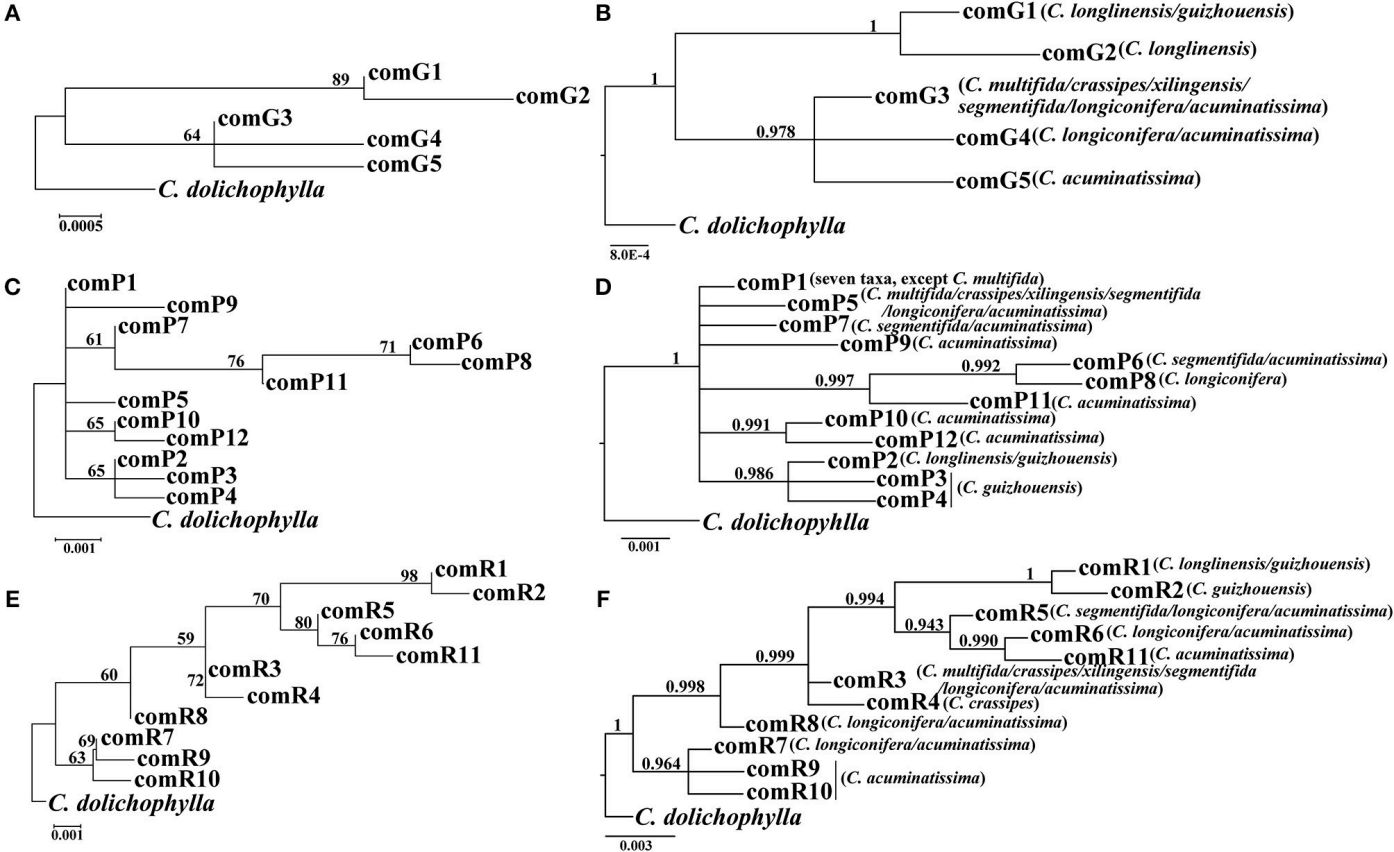
**Phylogenetic trees of nuclear haplotypes based on (A,C,E) Maximum Likelihood (ML) and (B,D,F) Bayesian Inference (BI) methods (*GTP*: (A,B); *PHYP*: (C,D); *PPRC*: (E,F)**. The number (if ≥ 50 or 0.5) on each branch indicates the **(A,C,E)** bootstrap value (BS) and **(B,D,F)** posterior probability (PP).

Our phylogenetic analyses showed no major conflicts among the ML and BI topologies with the ML tree showing more detailed internal evolutionary relationships within haplotypes (Figures 2, 3). All the plastid haplotypes inferred from the *C. segmentifida* complex were monophyletic and clustered together into two deep clades, one composed of plastid haplotypes from *C. guizhouensis, C. longlinensis* and the other clade consisted of *C. multifida, C. crassipes, C. xilingensis, C. segmentifida, C. longiconifera*, and *C. acuminatissima*. Thus, the result rejected the hypothesis that the *C. segmentifida* complex consisted of eight distinct taxa or one single lineage. These two assemblages detected by plastid DNA were also supported by high bootstrap (BS = 96, 94, Figure 2A) and posterior probability (PP = 1, Figure 2B) values. Interspecific relationships were unresolved in all the nuclear gene analyses except *GTP*, which showed a consistent cladogram with plastid DNA, suggesting the hypothesis that two lineages maybe occurred in this complex (Figures 3A,B).

### NJ tree and structure inference

After calculating three common genetic diversity indices, 12 microsatellite loci were selected for delimitating species boundaries within the *C. segmentifida* complex. The 12 microsatellite loci identified 179 alleles in the *C. segmentifida* complex in total with a mean value of 14.917. The number of alleles (*N*_A_) ranged from 3 to 49, expected heterozygosity (*H*_E_) ranged from 0.056 to 0.953, and observed heterozygosity (*H*_O_) ranged from 0.051 to 0.662. The levels of genetic diversity estimated from 12 loci were different. The highest level of genetic diversity was detected in the locus Cha-estssr01, while the lowest level of genetic diversity was detected in the locus Cha05 (Table 3). The 12 polymorphic microsatellites were used to investigate the phylogenetic relationships of 19 populations from the eight taxa in *C. segmentifida* complex.

**Figure 4:**
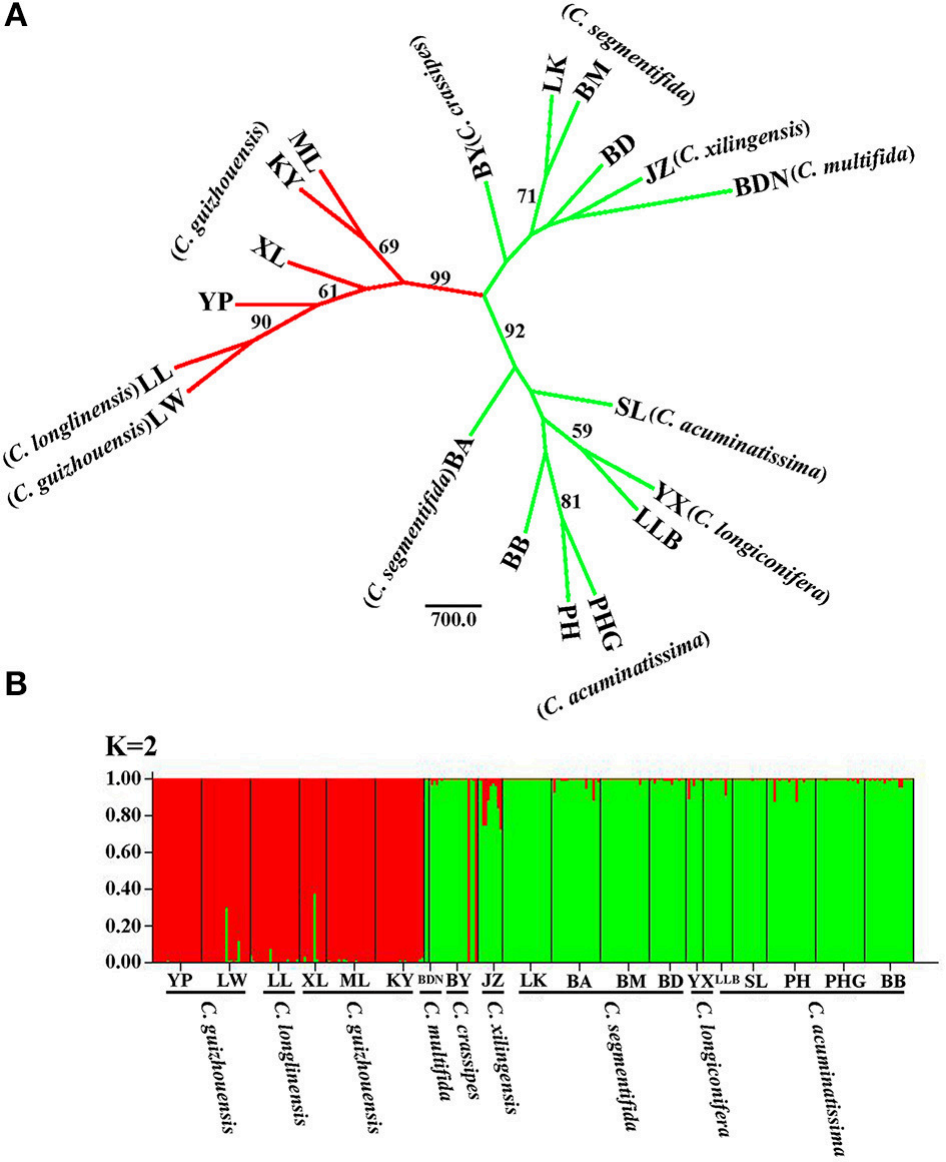
**(A)** The Neighbour-joining consensus tree (NJ tree) based on Nei's 1983 genetic distance and **(B)** Bayesian inference (*K* = 2) of microsatellite phenotype from 19 populations of 311 individuals of the *C. segmentifida* complex. Numbers (if ≥ 50) on branches indicated bootstrap values from 1000 replicates.

For the result of NJ tree, all populations of the *C. segmentifida* complex were grouped into three main clades with two clades supported by a high BS value (99/92) (Figure 4A). *Cycas longlinensis* together with five populations of *C. guizhouensis* were clustered into a clade, five populations of *C. acuminatissima* together with one population *C. segmentifida* as well as *C. longiconifera* were grouped into a clade and the remaining six populations from four taxa were grouped into another clade with relative low BS value. Although three clades were clustered in the NJ tree, only two lineages were revealed. The hypothesis of a two-lineage derived pattern could also be supported by the Structure analysis based on the ΔK method, the optimal K value was *K* = 2, showing that the 19 populations from the *C. segmentifida* complex were separated into two distinct clusters (Figure 4B). One cluster contained five populations of *C. guizhouensis* and the only one population of *C. longlinensis* and another harbored 13 populations of the remaining six taxa. Furthermore, the two clusters had less or no gene flow or introgression with each other through the results displayed by Structure analysis, implying that this complex contained only two evolutionary significant units with little contact.

## Discussion

### Species delimitation based on tree-based methods by DNA sequences

Tree-based species delimitation methods are on the basis of the concordance between some properties such as monophyly and geography with phylogenetic tree topologies. The tree-based methods by checking DNA sequences variations were formerly recognized and applied based on various strategies in delimitating boundaries of different species (Pérez-Losada et al., 2005; Perez-Losada et al., 2009; Rosell et al., 2010; Harrington and Near, 2012; Liu et al., 2015; Su et al., 2015; Tsai et al., 2015). According to the Wiens and Penkrot's criterion (Wiens and Penkrot, 2002), if haplotypes from the same locality fail to cluster together, it may suggest gene flow between localities (i.e., focal species = single species). However, the plastid haplotype and *GTP* haplotype trees showed that haplotypes of the focal species, *C. segmentifida* complex, were exclusive and there was no gene flow between their two basal lineages in this study. On one hand, for nuclear haplotypes' distributions in the *C. segmentifida* complex, *C. longlinensis* mainly shared the same nuclear haplotypes with *C. guizhouensis* and the two taxa owned unique nuclear haplotypes, indicating that *C. longlinensis* had closer relationship with *C. guizhouensis* than any other species. The remaining six taxa shared same nuclear haplotypes which were specific for them, indicating the remaining six taxa were more closely related. For plastid haplotypes' distributions in the *C. segmentifida* complex, *C. longlinensis* owned the same plastid haplotype comH1 with *C. guizhouensis* without its unique plastid haplotypes, indicating a possible identical common ancestor between *C. longlinensis* and *C. guizhouensis*. In addition, *C. multifida, C. xilingensis, C. crassipes*, and *C. segmentifida* only occupied and shared one single unique plastid haplotype comH6, suggesting the four taxa came from the same lineage, while *C. longiconifera* and *C. acuminatissima* derived their unique plastid haplotypes not shared with other taxa. On the other hand, *C. longlinensis* also shared closer geographical distance with *C. guizhouensis* in Nanpan River basin than other members of the complex which mainly distributed along the You River basin. Therefore, based on the phylogeny of plastid haplotypes and *GTP* nuclear haplotypes (Brower, 1999), two sets of topologically contiguous populations can be divided into two separate species. Our conclusion supports Wang's (Wang, 2000) previous morphological study and Ma's (Ma, 2005) research based on statistical analyses of morphological variations and UPGMA dendrogram revealed by ISSR markers, but conflicts with other treatments mainly based on morphological traits (Chen and Stevenson, 1999; Huang, 2001; Whiteloek, 2002).

The other two nuclear genes cannot deal with species boundaries of this complex as their haplotype phylogenetic trees showed no clear phylogenetic clades corresponding to one or more haplotypes from continuous populations. The congruence between taxonomy and plastid genes along with one of the nuclear genes *GTP* but not with two others may be caused by the specific history of the recently diversified *Cycas* which are still sorting their lineage processes by isolation or genetic drift. As nuclear genes own a higher effective population size (*N*e) that is four times higher than a given plastid gene, they can strongly influence the rate at which the haplotypes of a species become exclusive (Neigel and Avise, 1986; Moore, 1995). Therefore, on equal conditions, it takes four times longer for a nuclear gene to fix a species exclusive or distinct than its plastid genes (Moore, 1995). In addition, the disparate histories revealed by plastid and nuclear genes could be explained by the fact that the whole plastid genome is inherited all together, whereas recombination between chromosome and within chromosome usually drives to have different histories in the nuclear genes.

### Species delimitation based on NJ-tree and structure inference by microsatellites

It is believed that STRUCTURE analysis could cluster individuals regardless of their population-of-origin based on rough consistence to Hardy-Weinberg genetic expectations. The strategy employed by STRUCTURE is straightforward and matches reasonably well the properties of meta-population lineages (Shaffer and Thomson, 2007). Meanwhile, microsatellites can also have great probability of success resolving species boundaries largely because microsatellite markers have several ten times faster evolutionary rates than DNA sequences (Wolfe et al., 1987; Graur and Li, 2000; O'Connell and Ritland, 2004; Kuchma et al., 2011). In this study, both the NJ tree and STRUCTURE analysis derived from microsatellite data revealed the same clustering for the *C. segmentifida* complex, two major lineages, one contained all populations of *C. longlinensis* and *C. guizhouensis* while the other included all populations of the remaining six taxa, which were in accordance with the above conclusion uncovered by DNA sequences. It is noteworthy that the NJ tree revealed two subclades in the “*C. segmentifida*” (BND-BB) clade. Both the two subclades occupied populations of *C. segmentifida*, suggesting slight or recent differentiation among populations within the BND-BB clade, while the differentiation was not enough for the two subclades to sort to morphological distinct species.

Combining evidences from nuclear and plastid DNA sequences, microsatellites analysis, as well as the geographical distribution, we propose the *C. segmentifida* complex to be divided into two exclusive species: *Cycas guizhouensis* K. M. Lan et R. F. Zou and *Cycas segmentifida* D. Y. Wang et C. Y. Deng. Our species delimitation within this complex can offer guidelines for further morphological taxonomic revision and conservation studies.

### Taxonomic treatment

*Cycas guizhouensis* K. M. Lan et R. F. Zou in Acta Phytotax. Sin. 21(2): 209-210, 1983.

Synonym: *Cycas longlinensis* H. T. Chang et Y. C. Zhong in Acta Sci. Nat. Univ. Sunyatseni, 36(3): 68, 1997.

*Cycas segmentifida* D. Y. Wang et C. Y. Deng in Encephalartos, 43: 11-14, 1995.

Synonyms: *Cycas multifida* H. T. Chang et Y. C. Zhong in Acta Sci. Nat. Univ. Sunyatseni, 36(3): 70, 1997; *Cycas xilingensis* H. T. Chang et Y. C. Zhong in Acta Sci. Nat. Univ. Sunyatseni, 36(3): 69, 1997; *Cycas longiconifera* H. T. Chang, Y. C. Zhong et Y. Y. Huang in Acta Sci. Nat. Univ. Sunyatseni, 37(4): 6, 1998; *Cycas acuminatissima* H. T. Chang, Y. C. Zhong et Z. F. Lu in Acta Sci. Nat. Univ. Sunyatseni, 37(4): 6, 1998; *Cycas crassipes* H. T. Chang, Y. C. Zhong et Z. F. Lu in Acta Sci. Nat. Univ. Sunyatseni, 38(3): 121-122, 1999.

## Author contributions

XF carried out the molecular genetic studies, participated in the data analysis and wrote the manuscript. JL participated in collection of study materials and DNA extraction. XG designed the research and collected study materials. All authors read and approved the final manuscript.

### Conflict of interest statement

The authors declare that the research was conducted in the absence of any commercial or financial relationships that could be construed as a potential conflict of interest.
